# Supporting healthcare professionals to address child weight with parents: a qualitative study

**DOI:** 10.3399/BJGP.2023.0238

**Published:** 2024-04-03

**Authors:** Miranda Pallan, Frances Mason, Helen M Parretti, Peymané Adab, Sally Abbott, Kate Jolly

**Affiliations:** Institute of Applied Health Research, College of Medical and Dental Sciences, University of Birmingham, Birmingham.; Institute of Applied Health Research, College of Medical and Dental Sciences, University of Birmingham, Birmingham.; Norwich Medical School, Faculty of Medicine and Health, University of East Anglia, Norwich.; Institute of Applied Health Research, College of Medical and Dental Sciences, University of Birmingham, Birmingham.; Research Centre for Healthcare and Communities, Institute for Health and Wellbeing, Coventry University, Coventry; Department of Dietetics, University Hospitals Coventry and Warwickshire NHS Trust, Coventry.; Institute of Applied Health Research, College of Medical and Dental Sciences, University of Birmingham, Birmingham.

**Keywords:** child, general practice, paediatric obesity, primary health care, qualitative research, school nursing

## Abstract

**Background:**

Primary care and community healthcare professionals (HCPs) are well placed to discuss child excess weight with parents and support them to make changes. However, HCPs have concerns about addressing this issue. There is a need to understand the factors that influence HCPs in initiating these conversations to inform strategies to support them.

**Aim:**

To explore with HCPs, working in primary care and community settings, their experiences of having conversations about child weight with parents, and the factors that create barriers or facilitate them to have these conversations.

**Design and setting:**

A qualitative study with GPs, primary care nurses (PNs), and school nurses (SNs) in England.

**Method:**

GPs and PNs were recruited to participate in semi-structured interviews. SNs from a community healthcare NHS trust were recruited to participate in focus groups. Vignettes were used to stimulate discussion. Data were analysed guided by the Framework approach.

**Results:**

Thirteen GPs, seven PNs, and 20 SNs participated. The following three themes were identified regarding barriers to HCPs having conversations about child excess weight: structural and organisational; HCP related; and parent or family related. The themes identified for the factors that facilitate these conversations were: structural changes (for example, dedicated appointments, access to weight assessment data, joined-up working across agencies); HCP approaches (for example, providing appropriate dietary and physical activity advice); and HCP knowledge and skills (for example, enhancing HCPs’ general and weight management-related skills and knowledge of child weight management services).

**Conclusion:**

A range of barriers exist to HCPs addressing child excess weight with parents in primary care and community settings. Actions to effect structural changes and support HCPs in developing relevant knowledge and skills are required to overcome these barriers.

## Introduction

There has been increasing focus on the role of GPs and other primary care and community healthcare professionals (HCPs) in health promotion,^1^^,^^2^ including addressing childhood obesity. However, there is evidence that GPs have concerns about initiating conversations with parents about their child’s weight.^3^ Two systematic reviews of qualitative studies conducted with HCPs in high-income countries^4^^,^^5^ reported barriers to HCPs having these conversations, including lack of knowledge and competence, time and clear referral pathways, concern about the HCP–patient relationship, social and cultural factors, and limited ability to address child weight without the support of other agencies. There has been less research that more directly informs the strategies and a lack of guidance on the support required to facilitate HCPs in having conversations about child weight with parents. In a 2018 meta-synthesis of 13 studies, some factors that HCPs found helpful when discussing child weight were identified. These factors mainly related to approaches to interacting with families and tools to assess child weight.^4^

In England, there is a programme of routine assessment of child weight: the National Child Measurement Programme (NCMP),^6^ which has more than 90% coverage and provides body mass index (BMI) data on children aged 4–5 years and 10–11 years.^7^ Currently, these data cannot be routinely accessed in primary care,^3^ but there is an aspiration to provide this access in the future (communication from Public Health England; now the Office for Health Improvement and Disparities, November 2019) as part of the NHS Digital Child Health programme,^8^ which could assist in supporting HCPs to have conversations about child weight.

This study explored GPs’, primary care nurses’ (PNs’), and school nurses’ (SNs’) experiences of consultations with parents to address overweight and/or obesity in children of primary school age (4–11 years). The study aimed to gain a deeper understanding of barriers and facilitators to having these conversations to inform potential future strategies to provide support to HCPs. The role of routinely collected data to support this HCP activity was also explored.

**Table:** How this fits in

Healthcare professionals (HCPs) working in primary care and community settings are known to experience barriers in discussing child excess weight with parents. A qualitative study was conducted with GPs, primary care nurses, and school nurses to further explore these barriers and identify facilitating factors to inform recommendations for actions to support HCPs in addressing child weight with parents. Structural changes within primary and community care, joined-up systems and data sharing across agencies, and development of HCP knowledge and skills, through core training and continuing professional development, will enable HCPs to discuss child weight and provide advice to parents.

## Method

### Study design

A qualitative study was conducted with HCPs from November 2019 to March 2020. GPs and PNs were invited to participate in interviews, and SNs were invited to participate in focus groups.

### Participants and recruitment

Participants working with populations with high ethnic diversity and socioeconomic deprivation were purposively sampled.^9^ Advertisement to GPs and PNs took place through primary care networks and social media. Monetary compensation was provided for their time.

SNs were invited to participate through the School Health Advisory Service in Birmingham (a large, superdiverse city in England with high deprivation).^10^ SNs received a continuing professional development certificate for their participation. Participants provided written consent and completed a questionnaire to provide demographic and professional details. For GPs and PNs, practice postcodes were mapped to Index of Multiple Deprivation (IMD) deciles.^11^

### Data collection

One author conducted interviews with GPs and PNs, either face to face or by telephone, enabling in-depth exploration of participants’ experiences.^12^ Two authors conducted focus groups with SN teams at their work base, enabling group interaction and sharing of experiences among team members.^13^ The authors were positioned within health care and public health.

Interview and focus group discussion guides were developed by drawing on the published literature, clinical experience of the research team, and advice from the study steering group (comprising GP, PN, SN, paediatrician, and public health representatives), and additionally addressed issues specified by the public health organisation that funded the study (Public Health England; now the Office for Health Improvement and Disparities). The following two approaches were incorporated: 1) narratives from HCPs on their experiences; supplemented with 2) clinical vignettes,^14^ developed in consultation with an HCP advisory group (Box 1 and Supplementary Information S1–S4) to encourage deeper reflection on how participants would approach this sensitive issue. For each participant group, three vignettes were developed. Vignettes were discussed in the same way for all participants. Further information on vignette development and use is provided in Supplementary Information S4.

**Box 1.** Summary of interview and focus group discussion guides and vignettes

**Objectives**	**Interview guide for GPs and primary care nurses**	**Focus group discussion guide for school nurses**
Explore experiences of addressing overweight or obesity in children with parents	Can you talk through your experiences of conversations that you have had with parents or carers about their child’s excess weight in general? Can you tell me about any consultations you have had where the parents have brought up the issue of their child’s excess weight? Can you tell me about consultations when you have initiated a conversation with a parent to address their child’s excess weight? How do you feel about having these conversations with parents?	Can you share with the group your experiences of conversations that you have had with parents or carers about their child’s excess weight in general? Can you tell me about discussions you have had with parents and carers when the main reason for the meeting is their child’s excess weight? Can you tell me about times when you have initiated a conversation with a parent or carer to address their child’s excess weight? How do you feel about having these conversations with parents?
Explore how HCPs use or would use weight status data or National Child Measurement Programme (NCMP) data within their consultations to address a child’s weight	How do you recognise that a child has excess weight? What information about a child’s weight status is available to you?	How do you recognise that a child has excess weight? How do you use the National Child Measurement Programme (NCMP) data?
Explore HCPs’ engagement with, perceived access to, and value of, relevant training opportunities	Have you been on any training to support you in having these conversations? What (other) training are you aware of that would help support you having conversations with parents about their children having a healthier weight?	Have you been on any training to support you in having these conversations? What (other) training are you aware of that would help support you having conversations with parents about their children having a healthier weight?
Explore how HCPs can best be supported to have consultations about healthier weight in both scenarios	Do you have any ideas about what might help you to have these conversations with parents? Can you identify any information you would like to have to support you having healthier weight conversations with parents? What skills do you think you need in order to have these conversations with parents and support them to change their child’s or family eating and physical activity behaviours? [Discussion of vignettes]	Do you have any ideas about what might help you to have these conversations with parents? Can you identify any information you would like to have to support you having healthier weight conversations with parents? What skills do you think you need in order to have these conversations with parents and support them to change their child’s/family eating and physical activity behaviours? [Discussion of vignettes]
Identify barriers and facilitators to having these consultations and potential recommendations for action	[Data arising from questions stated above and related prompts] [Discussion of vignettes]	[Data arising from questions stated above and related prompts] [Discussion of vignettes]
**Vignettes**		
1	*Child and parent consult about a non-acute health problem of the child; healthcare professional notices the child is overweight*	*Parent makes contact in response to NCMP feedback letter saying that the child has excess weight. The parent is not happy and does not agree that their child is overweight*
2	*Parent consults about their own health issue and the healthcare professional notices the accompanying child is overweight*	*Child and parent attend for management of a long-term condition (for example, asthma); healthcare professional notices the child has excess weight*
3	*Parent and child consult about a health problem of the child. Healthcare professional notices the child is overweight; there are social and/or cultural contexts that need to be considered*	*Child and parent or carer attend for a safeguarding health assessment. Child is assessed and has excess weight*

*HCP = healthcare professional.*

Interviews and focus groups were audio-recorded and transcribed. Throughout data collection, transcripts were reviewed (by two authors) and sufficient information power^15^ was judged to be reached after 20 interviews and three focus groups. One author kept a research journal during data collection that informed analysis.

### Data analysis

Analysis was conducted using NVivo (version 12) software and guided by the Framework method.^16^ An initial high-level framework was defined. Transcripts were coded using inductive thematic analysis^17^ and situated within the high-level framework. Two transcripts were coded independently by two authors and discussed to develop initial codes. Other transcripts were coded by one author. The data were charted to develop a framework matrix, which enabled identification of themes and subthemes. These were reviewed by the research team.

## Results

Thirteen GPs and seven PNs from seven different local authority areas in the West and East Midlands regions of England participated in interviews (average duration 50 min). Twenty SNs participated in three focus groups (average duration 62 min). Participant characteristics are presented in Table 1.

**Table 1.** Participant characteristics

		**All** (***N* = 40)**	**GPs (***N* **= 13)**	**Primary care nurses (***N* **= 7)**	**School nurses (***N* **= 20)**
Sex, *n* (%)	Female	36 (90.0)	9 (69.2)	7 (100)	20 (100)

Age, years, *n* (%)	20–39	15 (37.5)	6 (46.2)	1 (14.3)	8 (40.0)
	40–59	23 (57.5)	7 (53.8)	5 (71.4)	11 (55.0)
	≥60	1 (2.5)	0 (0.0)	0 (0.0)	1 (5.0)
	Missing data	1 (2.5)	0 (0.0)	1 (14.3)	0 (0.0)

Ethnic group, *n* (%)	White/British	24 (60.0)	6 (46.2)	7 (100.0)	11 (55.0)
	Pakistani	3 (7.5)	2 (15.4)	0 (0.0)	1 (5.0)
	Indian	8 (20.0)	5 (38.5)	0 (0.0)	3 (15.0)
	Black Caribbean	3 (7.5)	0 (0.0)	0 (0.0)	3 (15.0)
	White/Black Caribbean	1 (2.5)	0 (0.0)	0 (0.0)	1 (5.0)
	Missing data	1 (2.5)	0 (0.0)	0 (0.0)	1 (5.0)

Number of years in post, *n* (%)	<3	13 (32.5)	3 (23.1)	4 (57.1)	6 (30.0)
	4–10	17 (42.5)	4 (30.7)	2 (28.6)	11 (55.0)
	≥11	9 (22.5)	6 (46.2)	0 (0.0)	3 (15.0)
	Missing data	1 (2.5)	0 (0.0)	1 (14.3)	0 (0.0)

Deprivation decile (IMD 2019) of practice or district, *n* (%)	1 (most deprived)	18 (45.0)	6 (46.2)	2 (28.6)	10 (50.0)
	2	7 (17.5)	1 (7.7)	0 (0.0)	6 (30.0)
	3	5 (12.5)	1 (7.7)	0 (0.0)	4 (20.0)
	4	4 (10.0)	2 (15.4)	2 (28.6)	0 (0.0)
	5	4 (10.0)	2 (15.4)	2 (28.6)	0 (0.0)
	6	1 (2.5)	1 (7.7)	0 (0.0)	0 (0.0)
	7–10 (least deprived)	1 (2.5)	0 (0.0)	1 (14.3)	0 (0.0)

Proportion of patients of minority ethnicity in practice population compared with national average, *n* (%)	Higher	9 (45.0)	8 (61.5)	1 (14.3)	N/A
	Similar	4 (20.0)	2 (15.4)	2 (28.6)	N/A
	Lower	6 (30.0)	3 (23.1)	3 (42.9)	N/A
	Missing data	1 (5.0)	0 (0.0)	1 (14.3)	

*IMD = Index of Multiple Deprivation.*

**Figure 1.**
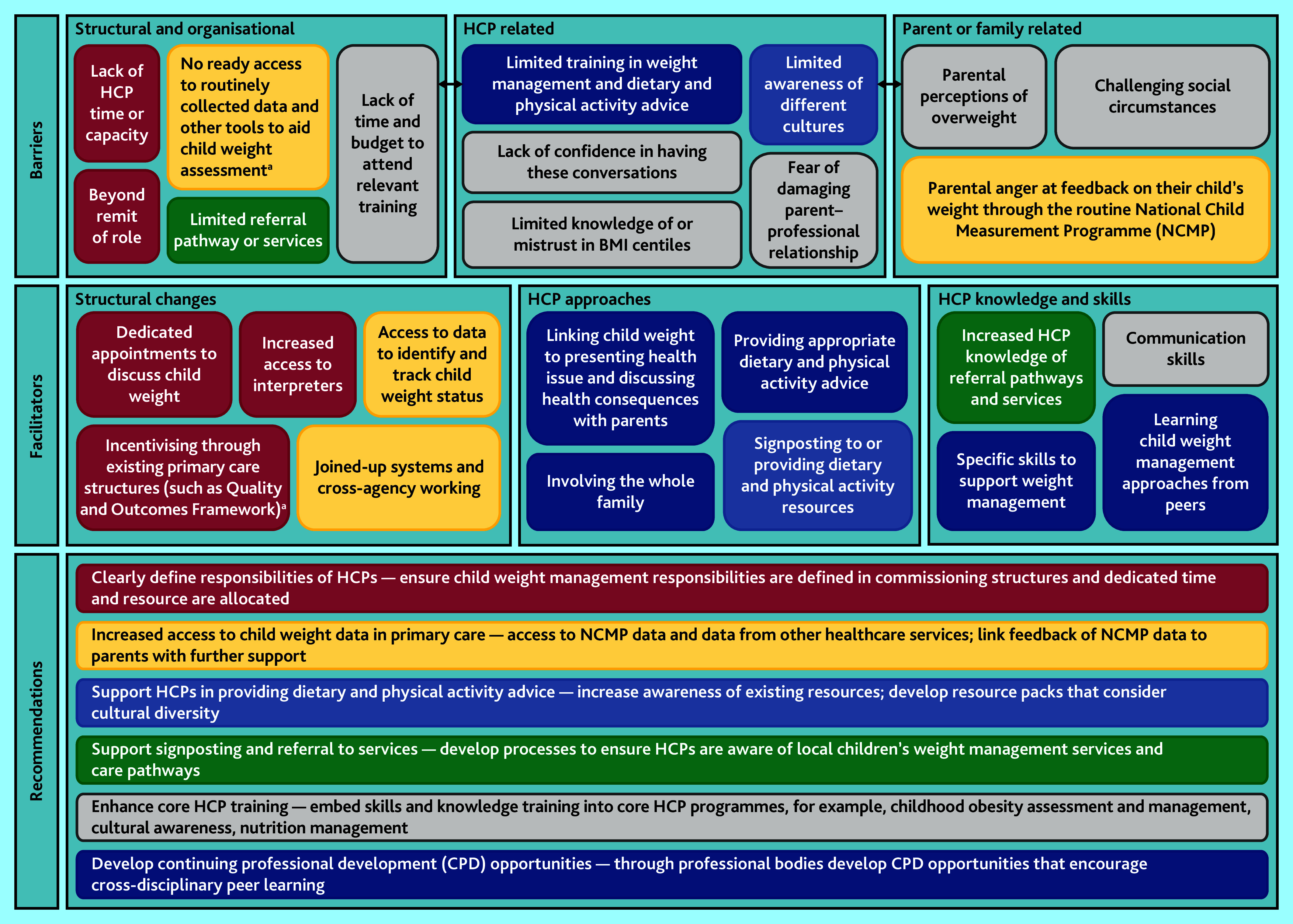
Barriers and facilitators to healthcare professionals having conversations with parents about child weight: themes and subthemes, and proposed recommendations for action. The boxes presenting each of the subthemes relating to barriers and facilitators are colour-coded to correspond to the recommendations that they have informed. ^a^GPs and primary care nurses only. BMI = body mass index. HCP = healthcare professional.

The following three themes were identified related to barriers to HCPs having conversations with parents about child weight: structural and organisational; HCP related; and parent or family related. A further three themes were identified related to facilitators to having these conversations, which were: structural changes; HCP approaches; and HCP knowledge and skills. The themes and related subthemes are presented in Figure 1 and additional quotes corresponding to each subtheme are presented in Supplementary Information S5.

### Barriers to HCP conversations with parents about child weight

#### Structural and organisational

Limited time and capacity were commonly cited as barriers, with HCPs identifying that dealing with the primary presenting issue left them with little time for opportunistic intervention. Although all participating HCPs felt well placed to address child overweight with parents, some felt that proactive child weight management intervention was beyond the remit of their role. SNs expressed that health promotion, such as addressing child overweight, was no longer in their role description and they were expected to focus on other priorities. Some GPs felt that addressing child weight was a marginal part of their role:

*‘Time, because usually they come in about something else. So, if you have got the worries about weight, often, we basically don’t have time.’*

(GP, 06)

*‘We’re not commissioned to do that any more. But then most of us feel that’s where our work lies.’*

(SN, focus group [FG] 01)

Although the HCPs recognised they had a role in providing child weight management advice, they felt limited in what they could offer, and the lack of referral opportunities deterred them from initiating conversations with parents:

*‘Unfortunately, we haven’t got access to a specific dietician for children for obesity. If you refer them, it tends to be rejected.’*

(PN, 06)

When asked about access to existing information on child weight status, GPs and PNs discussed that they had no access to these data from other sources (for example, the NCMP) and that, if they had ready access to it and tools to assist in overweight and/or obesity assessment, they could more easily address the issue with families. Many participants discussed the need to integrate data from different sources so weight could be longitudinally tracked in primary care, but acknowledged there was no current capability for this data integration:

*‘I think it* [NCMP data] *would be helpful … you’re* [then] *not just solely relying upon your visual recognition of whether the child is a little overweight or not, but actually you’ve got something there, and that would actually help the conversation with parents.’*

(GP, 01)

Some GPs and PNs discussed time and cost barriers to attend the training needed to help them have these conversations with parents (see HCP-related barriers):

*‘… most training is in the core working hours. But if you’re away then you have to put somebody* [in your place]*. Then it’s going to cost an arm and a leg to do that.’*

(GP, 04)

#### HCP related

Some GPs and PNs feared that raising the issue of child weight would damage their relationship with the parent, especially when parents were attending a consultation with a differing agenda. They felt that parents who were unprepared for a conversation about child weight could be defensive. Some HCPs did not feel confident in raising the issue, and this was sometimes related to the HCP living with overweight themselves. Another factor related to HCP confidence was their perceived low awareness of different cultures, both in terms of understanding of how childhood overweight may be viewed and providing culturally appropriate advice:

*‘If they* [the parents] *are quite standoffish already as it is … it’s quite hard to then start bringing up their child’s weight.’*

(GP, 05)

*‘… you can sometimes feel that the family might be a bit … they’re coming for a different thing and this was not on their agenda today. It was not on their radar.’*

(GP, 09)

*‘That’s* [cultural beliefs relating to child weight] *one of the things I’m struggling with and I’ll be brutally honest … It’s those cultural beliefs I’ve not got a handle on yet.’*

(GP, 03)

The use of BMI centiles by HCPs to assess overweight was identified as an issue. SNs had a good understanding of BMI centile charts, but some expressed mistrust in them, preferring to use height and weight centile charts to assess overweight. GPs and PNs were less familiar with BMI centiles, with some also perceiving that BMI is not a good measure for younger children:

*‘… we used to use the* [height and weight] *centile charts and actually the BMI will put a lot more children in an overweight category than the centile charts will.’*

(SN, FG01)

When asked about the skills and training needed, participants often highlighted the limited training that the HCPs receive on childhood obesity and nutrition, which contributed to reluctance to initiate conversations about child weight:

*‘And I think it’s a problem that lots of people would like to tackle but they just don’t really know where to start … we don’t have much in the way of training about childhood nutrition.’*

(GP, 07)

#### Parent or family related

HCPs identified that parents often do not perceive their children (or themselves) to have excess weight, which could be a barrier. In some cases, HCPs discussed this in the context of ethnic and cultural factors:

*‘I think, in this population the issue is that the certain minorities that we have here, what an ideal or a healthy weight is, isn’t a true reflection of what medically we think is a good weight.*’

(GP, 02)

During discussion of the vignettes, several HCPs acknowledged that challenging family circumstances (for example, low income, mental health challenges, and parental separation) may make them more reluctant to address child overweight:

*‘I think it’s quite difficult to talk about weight when they’ve* [the family] *got so much going on.’*

(SN, FG01; discussing vignette 3)

SNs particularly saw the feedback letter that parents of children with overweight and/or obesity receive following NCMP measurement as an issue. The SNs had a role in responding to queries raised by parents following receipt of the feedback letter and discussed that the wording was problematic, often causing anger and distress:

*‘I’ve had quite a few parents who were very upset receiving the* [NCMP feedback] *letter. I think it’s more the way the letter is worded.’*

(SN, FG03)

### Facilitators of HCP conversations with parents about child weight

#### Structural changes

One strategy identified to address lack of time was making dedicated appointments to discuss child excess weight. There was a sense that this also gave parents time to prepare for the conversation:

*‘It* [discussion about child weight] *requires more than the extra thirty seconds I have at the end of the ten-minute consultation. Sometimes I will get over that by saying, “I think we need to discuss your child’s general health, please book a separate appointment to do that.”’*

(GP, 02)

There was a perceived need for more cross-agency working. This related to the integration of data systems to enable HCPs to access and share health information and measurements for children with other healthcare organisations, and to joined-up working with schools and other agencies. When asked about which information would be useful to them, HCPs identified that access to and integration of routinely collected data from health services and the NCMP would enable them to easily track children’s weight status and be alerted to children who were rapidly gaining weight:

*‘ … it’s all this collaborative working isn’t it? It’ll stop doing the work twice … and also it’s about being on the same page as well because a parent may come to us and say, “I don’t know what the problem is”, and we can say, “Well actually, let’s have a look at what the school nurse measured.”’*

(PN, 03)

Through discussion of the vignettes, access to interpreters for parents with limited English was identified as a facilitator to having conversations about child excess weight in these families. When asked directly about incentives, some but not all GPs identified that incentives through the Quality and Outcomes Framework (remuneration to general practices for specified healthcare activities) to proactively address childhood excess weight could be a useful approach. However, some HCPs felt that this may result in a ‘tick-box’ approach:

*‘If the patient spoke limited English but was able to understand some of what I’m saying, I would probably address the main symptom … and then invite them to return with an interpreter for the follow-up appointment.’*

(GP, 10; discussing vignette 1)

*‘… if you want it done* [GPs to have conversations with parents about child excess weight]*, you have to attach some kind of incentive for all GPs to take it seriously, unfortunately. But at the same time I think there runs the risk of it becoming a tick-box exercise, to get that payment.’*

(GP, 02)

#### HCP approaches

HCPs identified several useful approaches to conversations with parents about child excess weight. They described linking weight to the problem that the child was presenting with and giving common-sense advice relating to food provision, diet, and physical activity. When training and skills were discussed, some highlighted that more training on giving this advice would further facilitate them having these conversations (see HCP knowledge and skills). Many HCPs recognised the need to involve the whole family when providing this advice:

*‘I would relate it to the problem that they presented with so, “One of the other things that might be a factor here might be that the child is carrying more weight than they should be. And that’s something that you might want to tackle.”’*

(GP, 07)

*‘I had one mum and her child was overweight, but she was a young parent and she actually didn’t know how to cook the dinners and, yeah … we spent a lot of time with her giving her worksheets, how to cook, make potato and beans rather than going to the fish and chip shop.’*

(SN, FG03)

*‘… it’s kind of addressing it in the sense of, can they do things as a family together in terms of their diet, changing the chapatis from white flour to brown flour, simple things like that. Changing the rice to brown rice. Maybe everyone going out for a walk together or going to the park together.’*

(GP, 05; discussing vignette 1)

*“We obviously want to be working with the family and for them to feel that it’s something beneficial, not just to the children but to the whole family.”*

(PN, 02)

Participants discussed signposting or providing lifestyle and weight management resources to families, often highlighting free online materials such as Change4Life (a national campaign to improve family dietary intake and physical activity). Some participants felt that a more bespoke resource pack containing specific weight management advice for families would assist them in opportunistically addressing child excess weight:

*‘I think an information package to be able to give to a parent would be great … I think from a healthcare professional point of view, is to give them something to put in their hands, that they will sit and look at when they’ve got five minutes.’*

(PN, 07)

#### HCP knowledge and skills

The need for increased HCP knowledge of child weight management services and the requirement to develop certain skills were identified as facilitators. Some HCPs in primary care perceived that there were existing services in their local areas that they could refer to, but it was not always known what these were:

*‘… offering people services that are available to them, if you know what the services are that are out there, then it makes the conversation a lot easier.’*

(GP, 01)

When asked about skills and training, participants identified generic HCP skills that were helpful to them in having conversations with parents about their child’s weight, including communication skills and being trained to have difficult conversations. Participants also identified useful specific skills, which included skills related to helping patients change behaviour (motivational interviewing), and to providing child weight management and healthy eating advice. Many participants felt that more training on providing this advice would help them address child weight. Some also commented on the need to consider different cultural contexts when providing healthy eating and weight management advice:

*‘I think — what’s the term? — motivational interviewing. I’ve done a lot of that in the past. I think that’s useful, looking at motivation to change.’*

(PN, 02)

*‘I’ve never really had any formal training on dealing with obesity in children … I think more practical training* [is needed] *on what you can actually offer and what is proven to work most effectively.’*

(GP, 02)

*‘You need to be aware of what different types of foods for different types of people* [referring to different cultures]*.’*

(SN, FG02)

Several PNs and SNs discussed the value of learning skills and approaches to having conversations and providing weight management advice from peers:

*‘What I actually believe as well is sharing the experiences as well; like, you say, “I did this with that one.”’*

(SN, FG02)

## Discussion

### Summary

GPs, PNs, and SNs recognised the importance of having conversations with parents about child excess weight but they experienced several barriers. While some related to family factors, many were structural or related to the HCPs themselves. The study was able to identify facilitating factors for HCPs in having these conversations, which have informed the potential strategies the authors have proposed (see Implications for practice) that would address both HCP-related and structural barriers within primary and community care. The study has provided valuable new insights on facilitating factors for HCPs, particularly around structural aspects of health care and supporting the development of HCPs’ knowledge and skills, as previous research has focused on facilitators relating to HCP–parent interactions.^4^

Structural changes that would support HCPs in having conversations with parents about child weight relate to organisational aspects within primary and community care (for example, system of dedicated appointments, better access to interpreters, and financial incentivisation). However, the need for a more joined-up approach across healthcare organisations and schools, with cross-agency working and sharing of information and weight-assessment data (including from the NCMP) was highlighted.

Several approaches that HCPs were already using were identified as good strategies when having conversations with parents about child weight, but participants clearly articulated where they needed further development in their knowledge and skills. This included knowledge of local child weight management provision, and skills in weight management advice and supporting behaviour change. Awareness of the cultural context and the ability to provide culturally appropriate advice were seen as important.

### Strengths and limitations

This study addressed a knowledge gap relating to strategies to support HCPs in having conversations with parents about child weight. The main primary and community care HCP groups that are well placed to address weight in primary school-aged children were included. Participants serving socioeconomically disadvantaged and ethnically diverse populations were recruited, which enabled the study to explore the challenges faced within these communities. The data collection approach included the use of vignettes, which is identified as a valuable method to explore in more depth the challenges faced by HCPs.^14^ This was further strengthened by the involvement of a multidisciplinary HCP group, who advised on the development of the discussion guides and vignettes.

Limitations include the focus on the English healthcare context. It is not clear whether these findings would be transferrable to the wider UK and international context; however, given the resonance of the findings with the international literature on the barriers to HCPs having these conversations,^4^^,^^5^ it is likely that there would be some applicability of the findings to other contexts. The recruitment approach for primary care professionals depended on advertising so the study is likely to have recruited participants with an interest in childhood overweight. While differing perspectives from other HCPs may have been missed, the interest of participants in this topic area helped in terms of exploring potential strategies to facilitate HCPs to address child weight. The SN participants were from one healthcare organisation, and so SN experiences may have been influenced by the local organisational context, limiting generalisability. However, this recruitment method allowed focus group methodology to be used, which enabled development of participants’ views through discussion with peers.^18^ The discussion guides were developed to ensure specific areas that were requested by the funders were covered, which may have influenced the data collected; however, it has been highlighted in the results where data were obtained in response to specific questions.

### Comparison with existing literature

Many of the barriers to HCPs having conversations with parents about child weight that were identified in the present study have been reported in other studies with HCPs,^4^^,^^5^ some of which have been in communities with high deprivation.^19^^,^^20^ In line with the present study’s findings, barriers identified in these latter studies included a lack of confidence in initiating discussions, inadequate time, fear of undermining parental trust, lack of recognition of overweight and cultural perceptions of weight by parents, difficulties in and avoidance of giving nutrition advice, and lack of collaborative working with other organisations.

HCPs’ fears of damaging parental trust and relationships, while common, may be unfounded. Adults with obesity mostly welcome health professionals initiating conversations about weight,^21^ and parents of young children are generally receptive to HCPs discussing child weight.^22^^,^^23^ However, the insights of this study’s participants into barriers, such as limited cultural awareness and a lack of training and skills in giving weight management advice, have reinforced previously identified HCP training gaps. The need to better address cultural competence and safety in GP and nurse training has been highlighted,^24^^,^^25^ as has the need for better skills development of medical and nursing professionals in nutrition, and obesity assessment and management.^26^^–^^29^ The development of skills and resources to support HCPs in child weight management activities was a prominent facilitating theme in the present study. In line with this, a study with English primary care nurses identified training and support in obesity management to be a factor in legitimising their role in undertaking weight management activities.^30^ Another factor highlighted was the importance of clarity around the role of the HCP in providing weight management advice, which again resonates with the findings of the present study. Qualitative studies with parents of children with excess weight have also shown that parents want practical and well-informed advice and resources from HCPs to help them manage their child’s weight.^23^^,^^31^

The present study has highlighted the need for awareness of children’s weight management services and joined-up approaches across agencies, including data sharing. A UK-based study exploring adult weight management services and their relationship with primary care identified that there is a need for services to develop better communication and relationships with primary care professionals.^32^ The present findings of the lack of HCPs’ knowledge of children’s weight management services would suggest that there is even greater need to improve communication between primary care and these services. In England, few children have BMI recorded in their primary care health record^33^ and there is evidence that HCPs are not good at identifying children with excess weight using observation alone.^34^ The NCMP provides objective data with high national coverage,^7^^,^^35^ therefore the integration of these data into primary care records represents a key opportunity in supporting HCPs to address child weight with parents. The present findings have also suggested that, in addition to NCMP data, sharing of information between primary care and other health services would enable more comprehensive engagement with child weight management.

### Implications for practice

The study has identified several recommendations for actions to address the identified barriers. The recommendations have been informed by the identified facilitators to support primary care and community HCPs to initiate conversations with parents and provide weight management advice and support to families. Figure 1 presents these recommendations, and the colour-coding shows which subthemes have informed each recommendation.

Clarity around the responsibilities of HCPs is needed. To achieve this clarity, responsibilities need to be defined within primary and community care commissioning arrangements, with adequate time and resource allocated to enable these responsibilities to be fulfilled. To assist in identification and tracking of child excess weight, the sharing of weight assessment data from both the NCMP and other health services with primary care is required. Access to NCMP data in primary care may also enable a more supportive feedback process of NCMP data to parents. Ready access to weight management resources that could be given to families would support HCPs in the initial management of child excess weight and it would be important to address cultural diversity in the development of new resources. In addition, there needs to be a more systematic approach to enabling HCPs to signpost and refer to existing weight management services through better communication processes between these services and primary and community care. Finally, the identified training gaps need to be addressed. This should be done through enhancing core HCP training by the inclusion of generic skills training, such as cultural awareness and competency, as well as more specific training relating to nutrition, and childhood overweight assessment and management. Additional training could be provided as part of continuing professional development programmes and incorporate peer and cross-disciplinary learning.

In conclusion, there are actions that could be taken to address the structural and HCP-related barriers to addressing child excess weight in primary and community care. These will require both national and local action to implement successfully.

## Data

Data (anonymised transcripts) could be made available on reasonable request to the authors.
